# JNK Contributes to Hif-1α Regulation in Hypoxic Neurons

**DOI:** 10.3390/molecules15010114

**Published:** 2009-12-30

**Authors:** Xanthi Antoniou, Alessandra Sclip, Cristina Ploia, Alessio Colombo, Gautier Moroy, Tiziana Borsello

**Affiliations:** 1 Istituto di Ricerche Farmacologiche "Mario Negri", Via La Masa 19, 20157 Milano, Italy; E-Mails: xanthi.antoniou@marionegri.it (X.A.); alessandra.sclip@marionegri.it (A.S.); cristina.ploia@marionegri.it (C.P.); alessiovittorio.colombo@marionegri.it (A.C.); 2 Xigen SA, Rue des Terreaux 17, CH-1015 Lausanne, Switzerland; E-Mail: gautier.moroy@univ-paris-diderot.fr (G.M.)

**Keywords:** JNK, HIF-1α, hypoxia, neurons

## Abstract

Hypoxia is an established factor of neurodegeneration. Nowadays, attention is directed at understanding how alterations in the expression of stress-related signaling proteins contribute to age dependent neuronal vulnerability to injury. The purpose of this study was to investigate how Hif-1α, a major neuroprotective factor, and JNK signaling, a key pathway in neurodegeneration, relate to hypoxic injury in young (6DIV) and adult (12DIV) neurons. We could show that in young neurons as compared to mature ones, the protective factor Hif-1α is more induced while the stress protein phospho-JNK displays lower basal levels. Indeed, changes in the expression levels of these proteins correlated with increased vulnerability of adult neurons to hypoxic injury. Furthermore, we describe for the first time that treatment with the D-JNKI1, a JNK-inhibiting peptide, rescues adult hypoxic neurons from death and contributes to Hif-1α upregulation, probably via a direct interaction with the Hif-1α protein.

## 1. Introduction

Hypoxia describes a pathological state where the brain or part of it is deprived of an adequate oxygen supply; such a situation can occur in acute (ischemia, traumatic brain injury) or chronic brain injuries (neurodegenerative diseases). 

Understanding the molecular mechanisms that render adult neurons, as opposed to young ones, more vulnerable to hypoxia is crucial to our understanding of brain injuries. Adult neurons are more vulnerable to hypoxia due to several factors, including a decreased ATP availability and alterations of the NMDA receptors system [1]. Recent investigations suggested that different protective factors such as neurotrophic factors (e.g BDNF) are downregulated in adult neurons, while stress proteins are upregulated [2]. In particular, evidence exist that neuronal specific genes (as opposed to other brain cells) are markedly downregulated with increased age, and this decrease is independent of neuronal loss [3,4].

Hypoxia inducible transcription factor 1-α (Hif-1α) is a crucial neuroprotective factor induced in conditions of reduced oxygen supply. Hif-1α was shown to be involved in a number of neurodegenerative disorders and its expression is decreased in adult mice brains, as well as in other tissues and cells [6,7]. In normoxic conditions, Hif-1α is degraded following hydroxylation of its proline residues PRO402 and PRO564, while in hypoxic conditions prolyl hydroxylases are inhibited thus Hif-1α accumulates in the cytosol, dimerises with its partner ARNT and translocates to the nucleus, where it induces transcription of different genes involved in energy metabolism, survival, and angiogenesis [8,9]. The pathways mediating Hif-1α are not fully characterised however, post-translational modifications including ubiquitination, SUMOylation and phosphorylation were all shown to participate in Hif-1 regulation [10,11].

Recent work indirectly suggested a role for the MAP Kinase c-Jun N-terminal kinase (JNK) in the regulation of Hif-1α. Comerford *et al*., [12] reported that JNK activation contributes to Hif-1α expression in hypoxic HeLa cells. Furthermore, c-Jun was shown to indirectly modulate Hif-1α [13].

In this study, we investigated the role of JNK signaling in the regulation of Hif-1α in young and adult neurons after hypoxic injury. For this purpose we used D-JNKI1, a cell permeable peptide previously shown to have protective effects on ischemia-induced neuronal death [14,15].

## 2. Results and Discussion

### 2.1. Differential susceptibility of young/immature compared to adult/mature neurons to hypoxic injury

Cortical neuronal cultures are an established model to study neuronal susceptibility to excitotoxic stimuli as a function of age. In our experimental setup, cortical neurons were cultured for 6 (6DIV) and 12 (12DIV) days in order to represent young/immature and adult/ mature fully differentiated neurons respectively [16,17,18].

**Figure 1:**
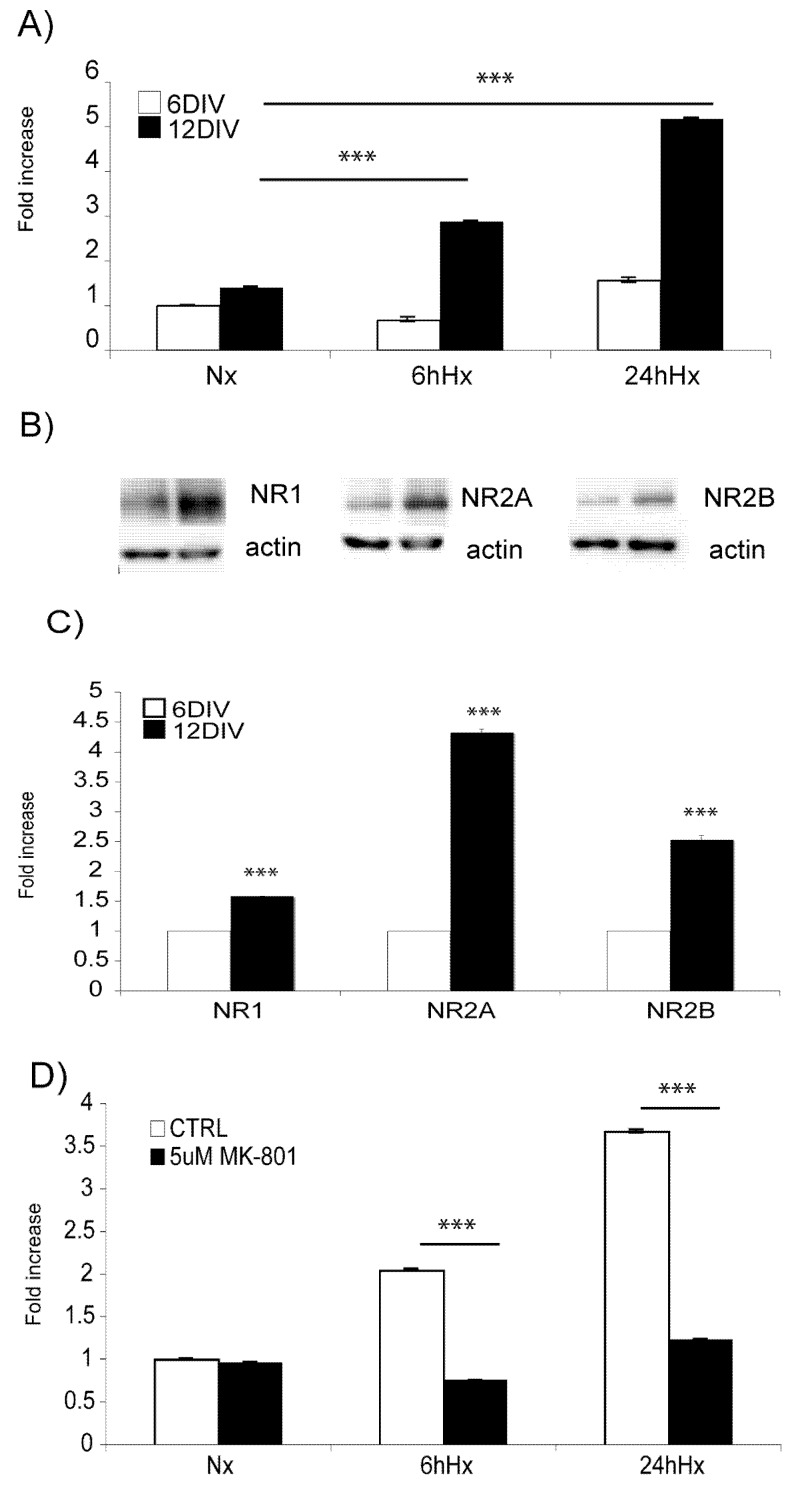
Characterisation of the model: A) 12DIV neurons are more susceptible to hypoxic injury. Hypoxia leads to a significant increase in neuronal death by 6h in adult neurons (p < 0.001). Young neurons are resistant to hypoxic injury for prolonged periods. B) Representative Western blot images of the NMDARs protein levels: NR1, NR2A, NR2B. C) Quantification of Western blots shows significant increase of all three receptor subtypes with increasing age (p < 0.001). D) Application of the NMDA blocker MK-801 (5 μM) rescues adult neurons from hypoxic neuronal death (p < 0.001). Data are presented as fold increase in comparison to normoxic untreated controls. Quantification is from three independent experiments.

Neuronal susceptibility to hypoxic insults was assessed by LDH assay (Figure 1A). Young neurons (6DIV) displayed resistance to 1% O_2_ up to 24 h. Differently, adult neurons (12DIV) revealed a time-dependent increase in neuronal death (1.87 ± 0.01 fold increase after 6h, p < 0.001; 4.17 ± 0.012 fold increase after 24h, p < 0.001 compared to normoxic neurons) following hypoxic insult. 

Following hypoxia, adult neurons displayed an increase in NMDA receptor protein levels as compared to younger-immature neurons. More specifically, Western blotting analysis confirmed that the NR1, NR2A and NR2B NMDA receptor subunits are higher in adult fully differentiated neurons as opposed to immature neurons (Figure 1B). A 0.59 ± 0.02, 3.32 ± 0.06, 1.53 ± 0.08 fold increase of NR1, NR2A and NR2B subunits’ protein levels respectively was observed (Figure 1C).

In addition, application of MK-801 (5 μM), a potent NMDA blocker, prevented hypoxia-induced neuronal death in adult neurons at both 6 h and 24 h (p < 0.001; Figure 1D) while having no effect on young ones (data not shown).

### 2.2. Basal levels of JNK signaling proteins increase with increasing age, while Hif-1α hypoxic induction decreases

**Figure 2:**
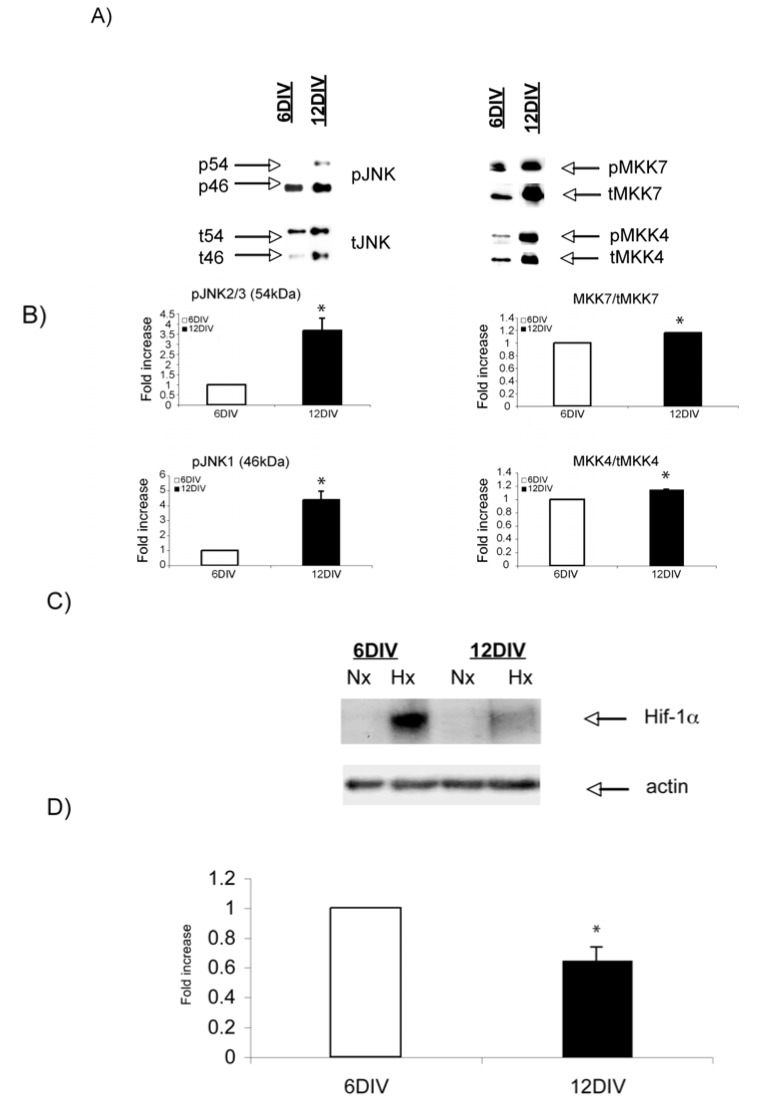
Basal levels of JNK signaling proteins increase with increasing age, while Hif-1α hypoxic induction decreases. (A) Representative Western blot images of phospho-JNK (pJNK), and its regulators pMKK7 and pMMK4 in 6DIV and 12DIV neurons. Basal levels of pJNK, pMKK7 and pMKK4 are higher in adult 12DIV neurons. (B) Quantification confirms significant up regulation of the JNK signaling components with increasing age (p < 0.05). (C) Representative Western blot images of Hif-1αhypoxic induction in 6DIV and 12DIV neurons. (D) Quantification of Western blots shows that Hif-1α hypoxic induction is 0.4 fold ± 0.086 lower in 12DIV adult neurons (p < 0.05). Data are presented as fold increase in comparison to normoxic controls. Quantification is from three independent experiments.

Increasing age leads to altered expression of specific stress-induced proteins. *In vitro*, increasing age led to an increase in the basal levels of the JNK signaling proteins, namely MKK7, MKK4 and JNK (Figure 2A). More importantly, the activation of the JNK cascade is more pronounced in mature compared to immature neurons (Figure 2A, Figure 2B, p < 0.05). On the other hand, Hif-1α levels are markedly reduced in adult hypoxic (1% O_2_ for 3 h) neurons compared to young ones as shown by Western blot analysis (Figure 2C) and subsequent quantification (0.4 fold ± 0.086 decrease, p < 0.05, Figure 2D).

### 2.3. Hypoxic regulation of Hif-1α and JNK signaling pathways

**Figure 3:**
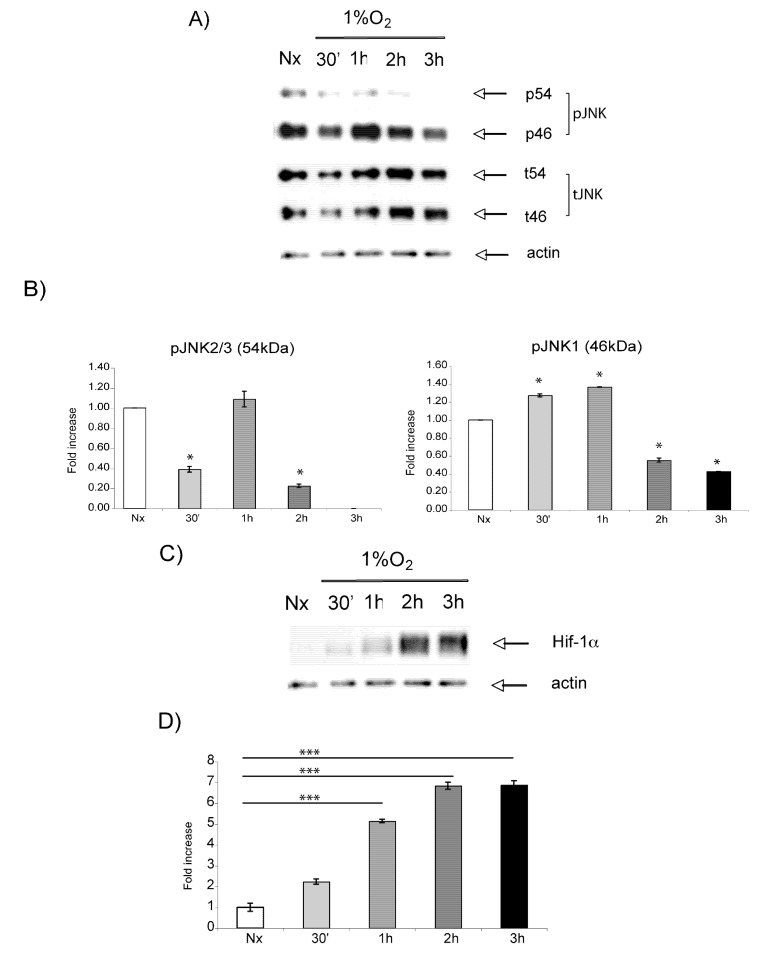
pJNK and Hif-1α are regulated by hypoxia at the protein level. A) Western blot analysis shows that pJNK is transiently induced by hypoxia, but falls below normoxic levels by 2h. B) Quantification confirms a biphasic regulation of pJNK2/3 (Significant downregulation by 30 min (p < 0.05)). pJNK1 was significantly increased by 30 min (p < 0.05). C) Western blot analysis reveals that Hif-1α expression is induced by hypoxia in a time-dependent manner. D) Quantification of Western blots shows that Hif-1α is significantly increased by 1h and reaches a peak by 3h (p < 0.001). Data are presented as fold increase in comparison to normoxic controls. Quantification is from three independent experiments.

As shown in Figure 1D, hypoxia is an NMDA-mediated signaling pathway. In view of the fact that JNK plays a pivotal role in NMDA-mediated excitotoxicity, we investigated its regulation in our hypoxic model. Western blot analysis revealed hypoxic regulation of pJNK (Figure 3A). Quantification analysis (Figure 3B) showed that pJNK2/3 (54 kDa) levels decreased by 30 min of hypoxic exposure to reach maximal levels of activation by 1 h. Yet, pJNK1 (46 kDa) levels were induced by hypoxia both at 30 min and 1 h (p < 0.05). A decline in the phosphorylation of all JNK isoforms was observed by 2 h and 3 h (p < 0.05). Differently, Hif-1α expression gradually increased to reach maximal levels by 3 h (Figure 3C, 3D). At this time point pJNK levels were markedly decreased. These data suggest an inverse correlation between JNK phosphorylation and Hif-1α expression.

### 2.4. A putative JNK binding domain on Hif1-α

In order to identify a possible direct interaction between JNK and Hif-1α we initially performed sequence analysis. JNK phosphorylates some of its targets through the JNK binding domain (JBD): K/RX_0-2_K/RX_0-4_L/I-X-L/I. A number of transcription factors, such as c-jun, ATF-2 and Elk-1 are modulated by JNK via a JBD interaction and are thus called JDB-dependent substrates [19].

**Figure 4:**
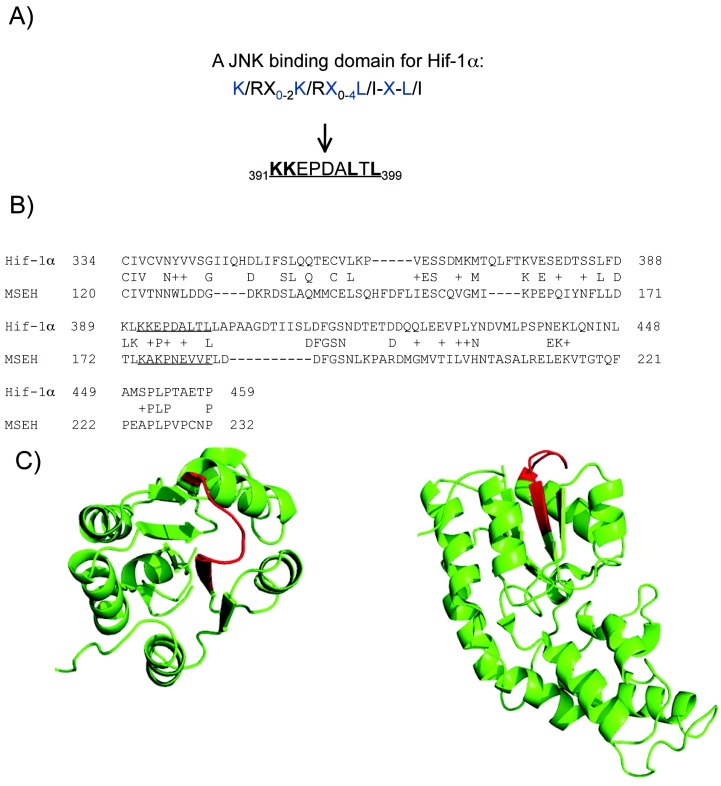
Computational evidence indicates Hif-1α as a potential target for JNK. A) Hif-1α contains a potential JNK binding domain (JBD) within the N-terminal VHL (Von Hippel-Lindau) recognition site. The JBD motif, as well the corresponding JBD sequence within Hif-1α are indicated. B) The local sequence alignment between MSEH and Hif-1α, the JBD motif and its corresponding sequence in MSEH are underlined. C) Cartoon representation of the 3D structure of MSEH (pdb code 1CQZ), the sequence corresponding to the JBD motif are coloured in red; at the left, view from the top and at the right, view from the side.

Sequence analysis of Hif-1α indicated the presence of a JBD motif within the N-terminal VHL (Von Hippel-Lindau) recognition site (Figure 4A). Although no protein in the PDB has good sequence homology with the whole human Hif-1α, some parts have low sequence similarity with known structures. Amongst them, the Murine Soluble Epoxide Hydrolase (MSEH) (pdb code 1CQZ) is the only one protein in the PDB that shares a local sequence similarity equal to 38.2%, with the human Hif-1α sequence containing the putative JBD (Figure 4B). Interestingly, the degree of similarity of 55.6% between the putative JBD and the corresponding sequence in MSEH is relatively high thus suggesting with reasonable confidence a structure similarity.

The structure of the MSEH shows that the sequence corresponding to the putative JBD forms a loop that is exposed to the solvent. Moreover, the secondary structures surrounding this sequence are sufficiently far not to disrupt the binding with other proteins, *i.e.,* the MSEH folding allows the interaction with another partner (Figure 4C). These observations support the hypothesis that this sequences maybe a binding domain for JNK.

### 2.5. D-JNKI1 treatment rescues hypoxic adult neurons from death

**Figure 5:**
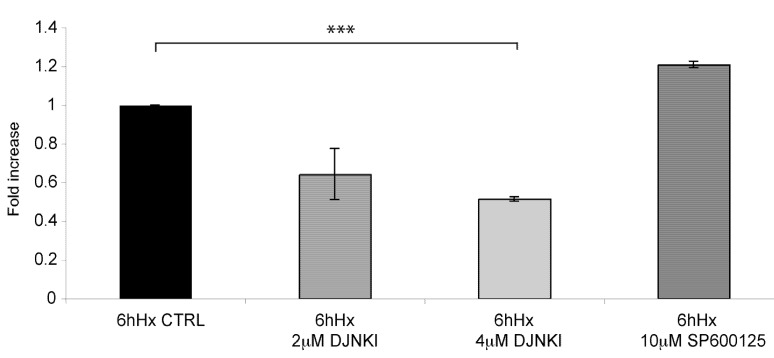
D-JNKI1 treatment rescues neurons from hypoxic induced death. D-JNKI1 treatment of adult neurons prior to hypoxic exposure led to a significant decrease in neuronal death, as assessed by LDH assay release. 2 μM D-JNKI1 led to a significant decrease of neuronal death, while 4 μM further decreased neuronal death (p < 0.001). Application of SP600125 (10 μM) prior to hypoxia did not revert neuronal death. Data are presented as fold increase in comparison to hypoxic controls. Quantification is from three independent experiments.

To elucidate the contribution of JNK signaling in hypoxic-induced neuronal death, neurons were treated with increasing D-JNKI1 concentrations and neuronal death was determined (Figure 5). Treatment with 2μM D-JNKI1 decreased neuronal death and 4μM D-JNKI1 led to a potentiation of this protective effect (p < 0.001). Application of SP600125, an ATP competitive and less specific JNK inhibitor, had no effect on the survival of the neurons.

We conclude that JNK signaling plays an important role in the early stages of hypoxia as underscored by the fact that inhibition of JNK led to a 55% survival against hypoxia as compared to hypoxic untreated controls. 

### 2.6. D-JNKI1 application increases hypoxia-induced Hif-1a levels

Hif-1α is an important neuroprotective factor in hypoxic/ischemic injury and it is strongly induced in this model (Figure 3C, Figure 3D). Since Hif-1α has a JBD and D-JNKI1 prevents hypoxia-induced neuronal death we investigated whether D-JNKI1 affects Hif-1α expression. 

**Figure 6:**
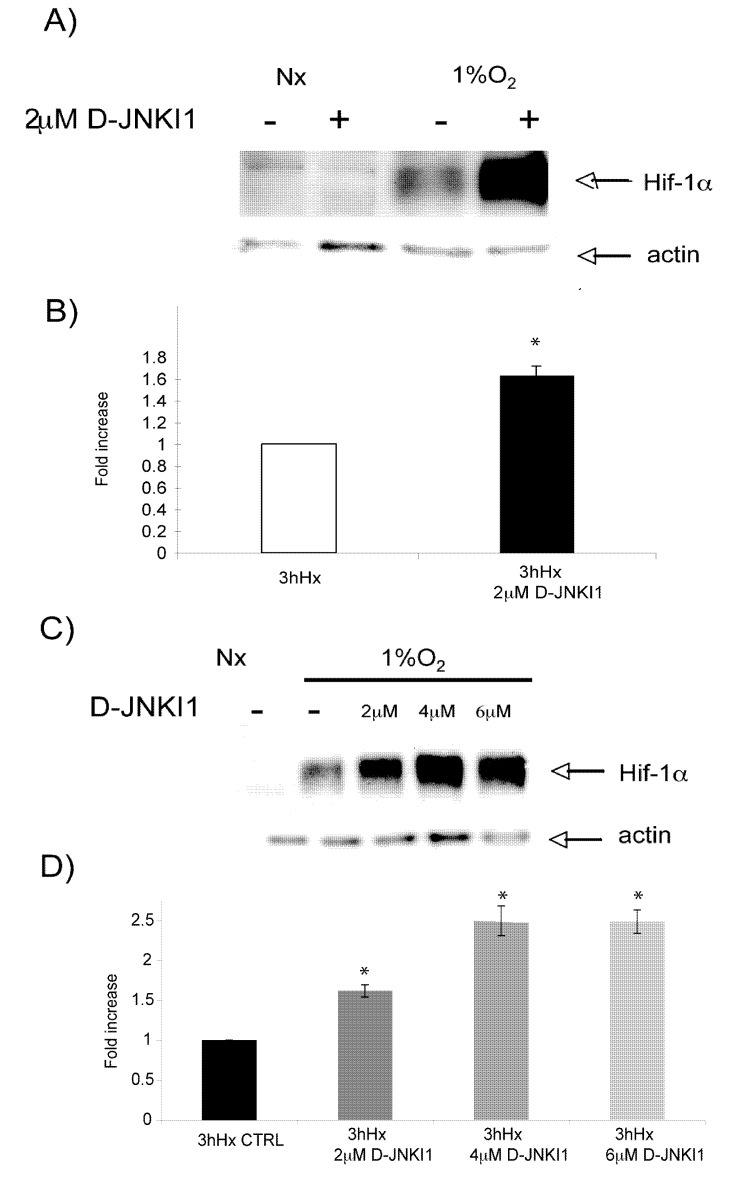
D-JNKI1 increases hypoxia-induced Hif-1αexpression. A) Representative Western blot of Hif-1α after hypoxic exposure shows D-JNKI1 (2 μΜ) increases hypoxia-induced Hif-1α expression. B) Quantification analysis confirmed that hypoxia-induced Hif-1α expression is significantly increased following D-JNKI1 treatment (p < 0.05). C) Representative Western Blot shows that hypoxia-induced Hif-1αexpression increases by D-JNKI1 in a dose dependent manner. D) Quantification confirmed significant upregulation of Hif-1α expression with increasing D-JNKI1 concentrations in comparison to hypoxic untreated controls (p < 0.05). Data are presented as fold increase in comparison to hypoxic untreated controls. Quantification is from three independent experiments.

Mature neurons were treated with D-JNKI1 and subsequently made hypoxic for 3h. Expression of Hif-1α protein levels were then determined by Western blotting. As expected Hif-1α expression was induced by 3 h of hypoxia. D-JNKI1 pre-treatment, led to a further increase in Hif-1-levels (0.61 ± 0.08 fold increase of Hif-1α induction in comparison to hypoxic controls, Figure 6A, Figure 6B). Treatment of neurons with increasing D-JNKI1 concentrations led to an additional increase of Hif-1α expression reaching a plateau at 4 μM (1.49 ± 0.18, p < 0.05), (Figure 6C, Figure 6D). Altogether, this data indicate that D-JNKI1 protects adult cortical neurons and this can partially modulate hypoxic-induced Hif-1α protein levels.

## Discussion

Excitotoxicity is a crucial factor in hypoxia, brain ischemia as well as being involved in a number of chronic neurodegenerative disorders [19]. Elucidating the pathways involved in excitotoxicity is crucial for the development of novel and more efficient therapies directed at treating brain injury. In line with the above, neuroprotection and brain repair in patients after brain damage are still major unfulfilled medical goals. 

Amongst other factors, age and degree of development influence injury outcome in neurons and thus should be considered as critical experimental variables so as to avoid misleading scientific outcomes [20]. For these reasons, we here analyze what are the factors that render adult neurons more susceptible to hypoxic injury in comparison to immature ones using an *in vitro* model of neuronal cortical cultures. A number of features seem to contribute to the enhanced vulnerability observed in adult neurons. Of those, alterations in cellular Ca^(2+)^ homeostasis is directly linked to an age-dependent component of vulnerability [23]. The data here reported, are in agreement with those of others who highlighted the intrinsic differences between immature and adult neurons in response to hypoxic injury [21,22]. 

We show that immature neurons display lower levels of NMDA receptor and that this correlates with their resistance to hypoxic injury. We then analyzed whether young and adult neurons display different basal and induced levels of Hif-1α (a neuroprotective protein) and JNK (a stress signaling protein). We here report an up-regulation in basal and inducible levels of JNK with increasing age (from 6DIV to 12DIV) *in vitro.* In line with this, expression of JNK activators MKK4 and MKK7 was also increased in mature neurons as compared to young ones. In accordance with our data, Jin *et al*., [23] reported upregulation of the JNK module in the aged hippocampus further supporting the idea that the JNK module positively correlates to aging and vulnerability. 

Differently, Hif-1α hypoxic induction decreased with age. Our data agree with those of others who showed reduced Hif-1α protein expression with increasing age in smooth muscle cells, rat cerebral cortex, mouse heart and carotid body [6,24]. Thus, although similar molecular pathways are employed in both young and adult neurons, it seems that the extent of their activation differs and probably contributes to the increased vulnerability of the latter to hypoxic stimuli. 

The exposure of neurons to oxygen-deprivation leads to the activation of both JNK and Hif-1 [25]. In fact, the pathways herein studied have been extensively explored in the field of neuroprotection and have been the targets of a series of therapeutical strategies [26]. In this investigation, a novel correlation between Hif -1α and JNK pathways is reported. Following BLAST analysis we identified a JBD domain within the Hif-1α sequence indicating Hif-1α as a possible target of JNK. Furthermore, we report that treatment with D-JNKI1, the Cell Permeable Peptide that specifically inhibits the interaction of JNK with its JBD targets, upregulates Hif-1α hypoxic induced expression in a dose-dependent manner. 

The transcription factor Hif-1α is the cellular oxygen sensor, its activation being essential for neuronal survival. Understanding the post-translational modifications that modulate Hif-1α activity can contribute to the design of novel therapeutical strategies aimed at treating hypoxic-related diseases. Modulation of Hif-1α hypoxic induction could be the outcome of a direct interaction with the JBD domain present in the Hif-1α sequence, or indirectly through regulation of c-Jun. In line with this, a study by Yu *et al*., suggested that c-Jun and not JNK can modulate Hif-1α activity in human cancer and microvascular endothelial cells [13]. Nevertheless, the presence of a JBD domain within the N-terminal VHL (Von Hippel-Lindau) recognition site would suggest that phosphorylation by JNK inhibits Hif-1α activation, although we cannot exclude the possibility that phosphorylation of Hif-1α by JNK might act upstream, and favors its degradation. Interestingly, Willam *et al*., [26], reported how peptides containing the sites of oxygen-regulated prolyl hydroxylation (Hif 390-417 and Hif 556-574) stabilise Hif-1α and induce Hif-1α signaling pathways. We did not observe Hif-1α stabilisation under normoxic conditions however; the incubation interval applied in this study might not have been sufficient to observe such a phenomenon. Further studies are required to explore this possibility.

We hereby report for the first time modulation of Hif-1α by JNK in adult fully-differentiated hypoxic neurons. More importantly, the D-JNKI1 treatment powerfully protected adult neurons against hypoxia and this correlated also with elevated Hif-1α levels. On the other hand the chemical JNK inhibitor SP600125 did not prevent neuronal death induced by hypoxia and this confirmed that the protection obtained by D-JNKI1 is mediated by the JBD domain.

## 3. Conclusions

The herein presented data underscore the crucial role played by Hif-1α and JNK signaling pathways in hypoxia-induced neuronal toxicity. Further, we clearly emphasize the potential of D-JNKI1 as a novel therapeutical strategy. This study provides a concrete platform to further investigate the modulation and interaction of Hif-1α and JNK signaling pathways with the goal of providing novel therapeutical targets to treat hypoxia-related neurological disorders.

## 4. Experimental

### 4.1. Neuronal cultures

Primary neuronal cultures were obtained from the cerebral cortex of two days post-natal rats, incubated with 200 units of papain (Sigma Aldrich) for 30’ at 34 °C, then with trypsin inhibitor (Sigma Aldrich) for 45’ at 34 °C and subsequently mechanically dissociated. All experimental procedures on live animals were performed in accordance with the European Communities Council Directive of 24 November 1986 (86/609/EEC) and all efforts were made to minimise animal suffering. Neurons were plated in 35 mm dishes (~7 × 10^5^ cells/dish) pre-coated with 25 μg/mL poly-D-lysine (Sigma Aldrich). The plating medium was B27/neurobasal (Life Technologies, Gaithersburg, MD) supplemented with 0,5 mM glutamine, 100 U/mL penicillin and 100 μg/mL streptomycin. The experiments were performed 6 and 12 days from plating date.

### 4.2. Hypoxic experiments and inhibitor treatments

Hypoxic experiments were performed in a glove-box chamber (*In vivo* 400, RUSKINN Technologies, Guiseley, UK) maintained at 37 °C with 5% CO_2_. Neurons were exposed to 1% O_2_ for 6 and 24 h and cell harvesting was performed within the chamber (*i.e.,* no reoxygenation of samples). Control cultures were maintained at 21% O_2_. Treatment with MK-801 (5 μM, Sigma), D-JNKI1 (2, 4, 6 μM, Xigen SA, Lausanne, Switzerland) or SP600125 (10 μM) was applied 45 minutes before hypoxic exposure. 

### 4.3. Lactate dehydrogenase assay

Neuronal death was evaluated by a lactate dehydrogenase (LDH) assay. LDH released into the culture medium was measured using the Cytotox 96 no radioactive cytotoxicity assay kit (Promega, WI). All LDH assays were performed in triplicate.

### 4.4. Cellular lysis

Total protein extracts were obtained by scraping cells in lysis buffer [27]. Cells were washed twice in ice-cold PBS and lysed for 20’ at 4 °C in 1% Triton x-100 lysis buffer supplemented with proteases (1x CPIK, Roche, 10634200) and phosphatases (1 μM 4-NPP, Roche, 10030536) inhibitors. 

### 4.5. Western blot analysis

10μg of total lysates were loaded and run onto 10% acrylamide gels and subsequently transferred to PVDF membranes. Incubation with primary antibodies was overnight at 4 ^o^C: NMDA NR1 (1:2,000 Invitrogen), NMDA NR2A (1:2,000 Invitrogen), NMDA NR2B (1:2,000 Invitrogen). Hif-1α (1:1,000 NOVUS), pJNK (1:1,000 Santa Cruz), JNK (1:1,000, Cell Signaling), pMKK7 (1:1,000 Cell Signaling), pMKK4 (1:2,000 Cell Signaling) tMKK7 (1:10,000 BD Transduction Laboratories), tMKK4 (1:1,000 Upstate), actin (1:5,000, Chemicon). Blots were developed using horseradish peroxidase-conjugated secondary antibodies and the ECL chemiluminescence system. All blots were normalized to actin (1:50,000 Millipore) and three independent experiments were performed. Western blots were quantified by densitometry using Quantity One software (Biorad).

### 4.6. Sequence analysis of the Hif-1α protein

The human Hif-1α sequence (number accession: Q16665) was downloaded from the UniProt website [28]. Its analysis was performed using a home-made script for highlighting the presence of putative JNK binding sequences characterized by the following sequence: K/R-X_0-2_-K/R-X_0-4_-L/I-X-L/I [19].

To allow the interaction with another partner, a binding sequence must be at the surface of the protein and not into the protein core. The knowledge of the three-dimensional structure is then crucial to know the position of the putative JNK binding sequence. Unfortunately, the structure of human Hif-1α is not available in the Protein Data Bank (PDB) [29], indicating that its structure has not been yet solved. A BLASTp research on the human Hif-1α sequence has been carried out in the PDB [30] in order to obtain structural data about human Hif-1α protein and, in particular, about its putative JNK binding site. The structures were visualized using Pymol program.

### 4.7. Statistical analysis

Graphs and analyses were performed using GraphPad Prism software. Statistical comparison among groups was made using one-way and two-way ANOVA tests. A value of p < 0.05 was considered significant. All results are expressed as mean ± standard deviation.
